# Crystal structure of the inverse crown ether tetra­kis­[μ_2_-bis­(tri­methyl­sil­yl)amido]-μ_4_-oxido-dicobalt(II)disodium, [Co_2_Na_2_{μ_2_-N(SiMe_3_)_2_}_4_](μ_4_-O)

**DOI:** 10.1107/S2056989016006861

**Published:** 2016-05-04

**Authors:** Christopher B. Hansen, Alexander S. Filatov, Gregory L. Hillhouse

**Affiliations:** a The University of Chicago, Department of Chemistry, 5735 S Ellis Ave., Chicago, IL 60637, USA

**Keywords:** crystal structure, μ_4_-oxido ligand, cobalt, inverse crown ether

## Abstract

The title compound, [Co_2_Na_2_{μ_2_-N(SiMe_3_)_2_}_4_](μ_4_-O), (I), represents a new entry in the class of inverse crown ethers. In the mol­ecule, each Co atom is formally in the oxidation state +II. The structure contains one half of a unique mol­ecule per asymmetric unit with the central μ_4_-oxido ligand residing on an inversion center, leading to a planar coordination to the Na and Co atoms. In the crystal, bulky tri­methyl­silyl substituents prevent additional inter­actions with cobalt. However, weak inter­molecular Na⋯H_3_C—Si inter­actions form an infinite chain along [010]. The structure is isotypic with its Mg, Mn and Zn analogues.

## Abstract 2

The first cobalt-containing inverse crown ether, [Co_2_Na_2_{μ_2_-N(SiMe_3_)_2_}_4_](μ_4_-O), features a central μ_4_-oxido ligand. Weak inter­molecular Na⋯H_3_C—Si inter­actions form an infinite chain extending along [010] in the crystal.

## Chemical context   

**Figure 1:**
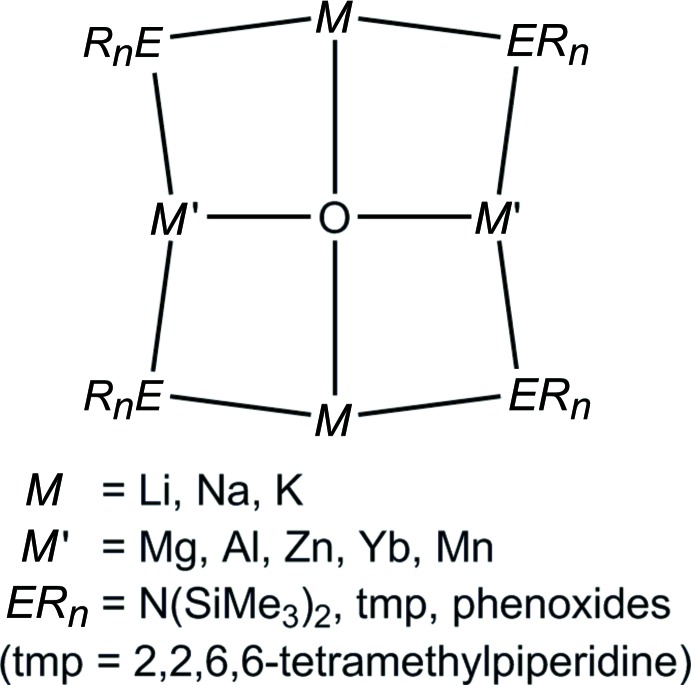
Schematic representation of inverse crown ethers that have previously been structurally characterized.

Compounds that feature oxido-bridged cobalt clusters have been of great inter­est in recent years as active homogeneous (Blakemore *et al.*, 2015 ▸) and heterogeneous (Kärkäs *et al.*, 2014 ▸) oxygen-evolution catalysts. Bridging cobalt-oxido species also find applications in magnetic materials (Heering *et al.*, 2013 ▸) and in hydro­carbon oxidation (Sumner & Steinmetz, 1985 ▸). In the course of studies of compounds with low-coordinate cobalt atoms (Hansen *et al.*, 2015 ▸), we have isolated and structurally characterized a cobalt-containing tetra­nuclear compound featuring a central μ_4_-bridging oxido ligand, [Co_2_Na_2_(μ_2_-N(SiMe_3_)_2_)_4_](μ_4_-O) (I). Compound (I) fits into the larger class of ‘inverse crown ethers’ illustrated in Fig. 1 ▸ (Mulvey, 2006 ▸).
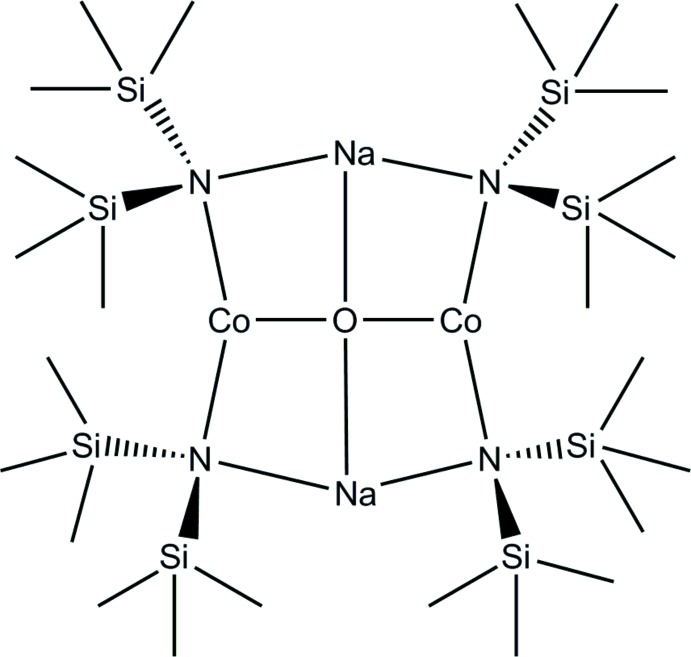


 Compound (I) is the first cobalt-based inverse crown ether. The majority of examples contain magnesium or zinc as *M*′, though manganese (Kennedy *et al.*, 2008 ▸; Mulvey *et al.*, 2010 ▸), aluminum (Wu *et al.*, 2010 ▸), and ytterbium (Lu *et al.*, 2010 ▸) complexes have been reported as well.

## Structural commentary   

**Figure 2:**
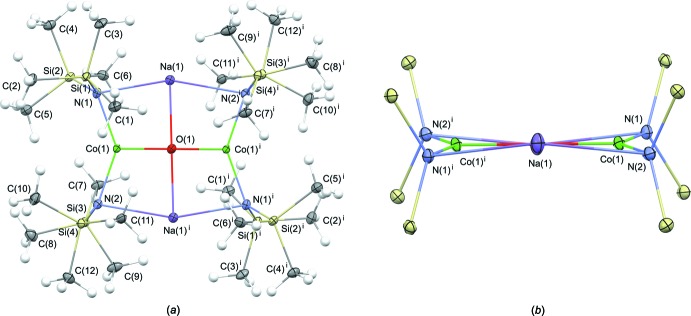
(*a*) The mol­ecular structure of (I), showing displacement ellipsoids at the 50% probability level. (*b*) An alternate view of (I) down the Na—O—Na axis displaying ring offsets. H and C atoms were truncated for clarity. [Symmetry code: (i) −*x* + 1, −*y* + 1, −*z* + 1.]

Crystals of (I) suitable for X-ray diffraction were obtained as reaction by-products *via* crystallization from toluene at 238 K. Attempts at a rational synthesis were not successful. The mol­ecular structure of compound (I) is shown in Fig. 2 ▸ *a* and relevant bond lengths and angles are presented in Table 1 ▸. The asymmetric unit contains half of a unique mol­ecule comprised of an oxygen atom located on an inversion center, one cobalt atom, one sodium atom, and two –N(SiMe_3_)_2_ ligands with the remainder of the mol­ecule being completed by application of inversion symmetry. Consequently, all opposing *M*—O—*M* angles (*M* = Co, Na) are crystallographically imposed to 180°. The four bridging nitro­gen atoms lie slightly out of plane from the four metal atoms, exhibiting a dihedral angle of 8.1 (2)° between their respective planes as shown in Fig. 2 ▸ *b*.

The majority of cobalt-bridging oxido compounds possess bent angles, so the μ_4_-oxido ligand in (I) is unusual in that it coordinates linearly to the opposing metal atoms. With a central oxido ligand, by charge balance each cobalt atom has formally an oxidation state of +II. While the paramagnetic nature of (I) prevents confirmation by NMR studies, it is unlikely that the central O atom is actually a hydroxido ligand. The structurally related anionic compound [Na_4_(μ_2_-N(SiMe_3_)_2_)_4_(μ_4_-OH)]^−^, which bears a central μ_4_-OH ligand, is noticeably pyramidalized, possessing Na—O—Na angles of 140.1 (2) and 142.4 (2)° (Clark *et al.*, 2009 ▸). Additionally, the Co1—O1 bond length of 1.8398 (9) Å in (I) is significantly shorter than those of other structurally characterized complexes of Co^II^ bearing approximately linear bridging hydroxido ligands, which display bond lengths ranging from 1.975 (2) to 2.3766 (6) Å (Li *et al.*, 2014 ▸; Reger *et al.*, 2014 ▸; Wendelstorf & Krämer, 1997 ▸).

The structure of compound (I) is isotypic with magnesium-, manganese-, and zinc-containing analogues of the general formula [*M*′_2_Na_2_(μ_2_-N(SiMe_3_)_2_)_4_](μ_4_-O), all of which contain planar linear bridging oxido ligands. Among the four compounds, (I) has comparatively short bonds. For instance, (I) displays the shortest *M*′—O [1.8398 (9) Å in (I) *versus* 1.8575 (4), 1.9272 (2), 1.8733 (9) Å in magnesium, manganese, zinc representatives, respectively] and shortest *M*′—N, [1.977 (4) and 1.980 (4) Å in (I) *versus* 2.054 (1) and 2.049 (1) Å (magnesium), 2.0909 (12) and 2.0884 (12) Å (manganese), and 1.986 (2) and 1.983 (2) Å (zinc)] bond lengths. The short bond lengths and acute bond angles may enhance the torsion of the metal plane from the nitro­gen plane.

## Supra­molecular features   

**Figure 3:**
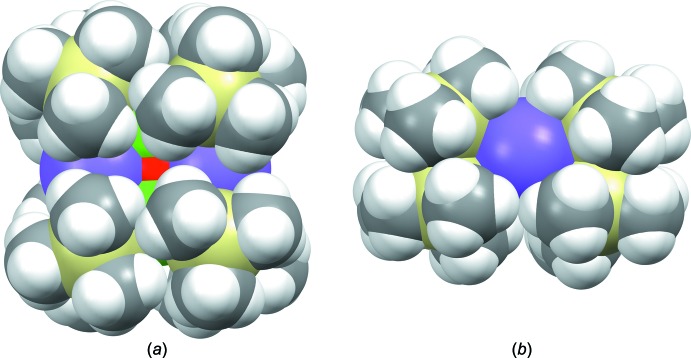
(*a*) Top view of a space-filling model of (I), showing the sterically shielded Co^II^ atoms. (*b*) Side-on view, displaying the open pocket around sodium that allows for weak inter­actions. [Color scheme: cobalt (green), sodium (violet), silicon (yellow), oxygen (red), carbon (gray), hydrogen (white)].

**Figure 4:**
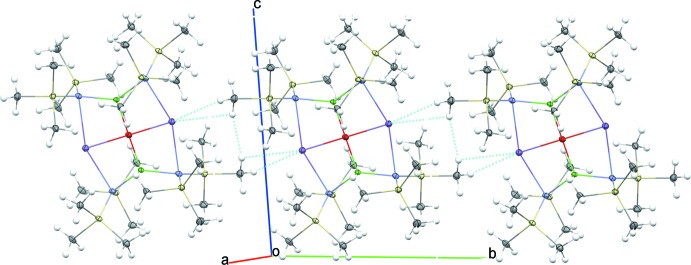
Packing diagram of (I), showing Na⋯H contacts forming an infinite chain that extends along [010]. (Symmetry code: −*x* + 1, −*y* + 1, −*z* + 1.)

In the solid state, the steric bulk of the tri­methyl­silyl­amide ligands prevents further inter­molecular inter­actions of either the cobalt atoms or the oxido ligand, as can be observed in the space filling model of (I) presented in Fig. 3 ▸ *a*. Some weak inter­actions can be noted for sodium, however, which is consistent with the open site around sodium visible in Fig. 3 ▸ *b*. The sodium atoms and one –Si—CH_3_ group from each mol­ecule coordinate to a neighboring –Si—CH_3_ group and sodium atom, respectively, forming an infinite chain extending along [010], as illustrated in Fig. 4 ▸. The two close Na⋯H contact distances of 2.961 and 2.886 Å fall within the range of previously structurally characterized literature examples of various mol­ecules containing sodium bis­(tri­methyl­sil­yl)amide moieties (2.55–3.0 Å). For selected examples, see: Driess *et al.* (1997 ▸); Sarazin *et al.* (2006 ▸); Kennedy *et al.* (2008 ▸). This type of inter­molecular inter­action has been previously noted in the solid state for related potassium-based inverse crown ethers bearing bridging peroxido ligands (Kennedy *et al.*, 1999 ▸), and in related sodium-containing precursors (Kennedy *et al.*, 2008 ▸).

## Database survey   

A search of the Cambridge Structural Database (CSD, Version 5.37, last update Nov. 2015; Groom *et al.*, 2016 ▸) reveals that structurally characterized oxido-centered inverse crown ethers are rare. The first examples were prepared from magnesium [CSD refcodes: EJEKEJ (Kennedy *et al.*, 2003 ▸); SUJQOD, SUJQUJ (Kennedy *et al.*, 1998 ▸)]. Further examples focused on zinc [CSD refcode: WOQTIF (Forbes *et al.*, 2000 ▸)], manganese [CSD refcodes: CIVRAB, CIVRIJ (Kennedy *et al.*, 2008 ▸); WUVROV (Mulvey *et al.*, 2010 ▸)], aluminum [CSD refcode: BABMEY (Wu *et al.*, 2010 ▸)] and ytterbium [CSD refcodes: IMIBUC, IMICUJ (Lu *et al.*, 2010 ▸)] complexes.

## Synthesis and crystallization   

Compound (I) was obtained as single crystals on multiple occasions as a side product of two different reactions; however, attempts at a rational synthesis were not successful. These reactions used conditions and reagents that were nominally free of oxygen and water. Nonetheless, trace oxygen or water are the likely sources of the bridging oxido ligand. Adventitious water (Lu *et al.*, 2010 ▸) and oxygen (Kennedy *et al.*, 2008 ▸) have both been shown to be potential oxygen-atom sources, and have been previously utilized to generate this type of structure. Additionally, fragmentation of tetra­hydro­furan has also been identified as a potential oxygen-atom source in one case (Mulvey *et al.*, 2010 ▸).

Method 1: In a glovebox [(IPr)CoCl_2_]_2_ (Matsubara *et al.*, 2012 ▸; Przyojski *et al.*, 2013 ▸) [IPr = 1,3-di(2,6-diiso­propyl­phen­yl)imidazolin-2-yl­idene] (50 mg, 0.048 mmol, 1 equiv.) was dissolved in 3 ml toluene and cooled to 238 K. A 238 K solution of NaN(SiMe_3_)_2_ (Sigma–Aldrich, titrated to 0.844M in THF) (22.9 µL, 0.193 mmol, 4 equiv.) was added dropwise to the solution of [(IPr)CoCl_2_]_2_ with stirring. The reaction mixture rapidly changed color from blue to turquoise to green and became turbid. The solution was allowed to warm to ambient temperature and stirred for 1 h. The reaction was filtered through Celite and the filtrate reduced to dryness under vacuum. The resulting green solid was dissolved in a minimal volume of toluene, passed through a Pasteur pipette filter, and stored at 238 K for several days. The resulting precipitate primarily consisted of thin green plates of (IPr)CoCl(N(SiMe_3_)_2_) (Hansen *et al.*, 2015 ▸), occasionally accompanied by a small number of dark green–blue blocks of (I).

Method 2: While attempting to prepare a compound of the type Na[Co(N(SiMe_3_)_2_)_3_], (I) was occasionally observed as a minor by-product during recrystallization attempts. In a typical reaction anhydrous CoCl_2_ (100 mg, 0.77 mmol, 1 equiv.) was suspended in 2 ml THF and cooled to 238 K. NaN(SiMe_3_)_2_ (423.6 mg, 2.31 mmol, 3 equiv.) was dissolved in 10 ml THF, cooled to 238 K, then added to the stirred slurry of CoCl_2_. The reaction mixture was allowed to warm to ambient temperature and stir overnight, over which time it slowly turned green and turbid. The reaction mixture was filtered through Celite and rinsed with additional THF until washings were colorless, leaving a white solid remaining on the Celite pad. The combined THF fractions were combined and concentrated under vacuum to a yield a waxy green solid. The resulting solid was recrystallized from a solution in a minimal volume of toluene cooled to 238 K. The title compound (I) was occasionally observed as blue–green blocks.

## Refinement   

Crystal data, data collection and structure refinement details are summarized in Table 2 ▸. All H atoms were placed at idealized positions with C—H = 0.98 Å, *U*_iso_(H) set to 1.5*U*_eq_(C). The initial structure solution and refinements had a goodness-of-fit of about 0.88 and many reflections with *F*_o_ > *F*_c_ suggesting possible twinning. The data reduction was revisited and the structure was refined under consideration as a two-component twin by non-merohedry. The second domain is rotated from the first domain by 3.3° about reciprocal axis [1 0 ½] as determined by *CELL_NOW* (Sheldrick, 2008 ▸). The twin ratio refined to a value of 0.88:0.12.

## Supplementary Material

Crystal structure: contains datablock(s) I. DOI: 10.1107/S2056989016006861/wm5287sup1.cif

Structure factors: contains datablock(s) I. DOI: 10.1107/S2056989016006861/wm5287Isup2.hkl

CCDC reference: 1476068

Additional supporting information:  crystallographic information; 3D view; checkCIF report

## Figures and Tables

**Table 1:** Selected geometric parameters (Å, °)

Co1—O1	1.8398 (9)	Na1—O1	2.314 (2)
Co1—N1	1.977 (4)	Na1—N1	2.579 (4)
Co1—N2	1.980 (4)	Na1—N2^i^	2.523 (4)
			
N1—Co1—N2	141.35 (17)	Co1—O1—Co1^i^	180.0
N2^i^—Na1—N1	155.82 (15)	Na1—O1—Na1^i^	180.00 (3)

Symmetry code: (i) 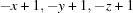.

**Table 2:** Experimental details

Crystal data	
Chemical formula	[Co_2_Na_2_O(C_6_H_18_NSi_2_)_4_]
*M*_r_	821.41
Crystal system, space group	Triclinic, *P* 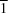
Temperature (K)	100
*a*, *b*, *c* (Å)	8.8839 (18), 10.591 (2), 12.700 (3)
α, β, γ (°)	96.75 (4), 108.93 (3), 99.15 (3)
*V* (Å^3^)	1097.4 (5)
*Z*	1
Radiation type	Mo *K*α
μ (mm^−1^)	1.02
Crystal size (mm)	0.30 × 0.24 × 0.20

Data collection	
Diffractometer	Bruker *SMART* *APEX* CCD
Absorption correction	Multi-scan (*TWINABS*; Bruker,2012 ▸)
*T*_min_, *T*_max_	0.57, 0.75
No. of measured, independent and observed [*I* > 2σ(*I*)] reflections	4421, 4421, 3107
*R*_int_	0.089
(sin θ/λ)_max_ (Å^−1^)	0.627

Refinement	
*R*[*F* ^2^ > 2σ(*F* ^2^)], *wR*(*F* ^2^), *S*	0.065, 0.154, 1.03
No. of reflections	4421
No. of parameters	200
H-atom treatment	H-atom parameters constrained
Δρ_max_, Δρ_min_ (e Å^−3^)	1.08, −0.54

Computer programs: *APEX2* (Bruker, 2014 ▸), *SAINT* (Bruker, 2013 ▸), *APEX3* (Bruker, 2015 ▸), *SHELXT* (Sheldrick, 2015*a* ▸), *SHELXL2014* (Sheldrick, 2015*b* ▸), *Mercury* (Macrae *et al.*, 2006 ▸) and *OLEX2* (Dolomanov *et al.*, 2009 ▸).

## supplementary crystallographic information

### Crystal data

**Table:**

[Co_2_Na_2_O(C_6_H_18_NSi_2_)_4_]	*Z* = 1
*M**_r_* = 821.41	*F*(000) = 440
Triclinic, *P*1	*D*_x_ = 1.243 Mg m^−^^3^
*a* = 8.8839 (18) Å	Mo *K*α radiation, λ = 0.71073 Å
*b* = 10.591 (2) Å	Cell parameters from 1020 reflections
*c* = 12.700 (3) Å	θ = 2.8–24.6°
α = 96.75 (4)°	µ = 1.02 mm^−^^1^
β = 108.93 (3)°	*T* = 100 K
γ = 99.15 (3)°	Block, green
*V* = 1097.4 (5) Å^3^	0.3 × 0.24 × 0.2 mm

### Data collection

**Table:**

Bruker SMART APEX CCD diffractometer	4421 independent reflections
Radiation source: sealed tube	3107 reflections with *I* > 2σ(*I*)
Graphite monochromator	*R*_int_ = 0.089
ω scans	θ_max_ = 26.5°, θ_min_ = 1.7°
Absorption correction: multi-scan (*TWINABS*; Bruker,2012)	*h* = −11→10
*T*_min_ = 0.57, *T*_max_ = 0.75	*k* = −13→13
4421 measured reflections	*l* = 0→15

### Refinement

**Table:**

Refinement on *F*^2^	Primary atom site location: dual
Least-squares matrix: full	Secondary atom site location: difference Fourier map
*R*[*F*^2^ > 2σ(*F*^2^)] = 0.065	Hydrogen site location: inferred from neighbouring sites
*wR*(*F*^2^) = 0.154	H-atom parameters constrained
*S* = 1.03	*w* = 1/[σ^2^(*F*_o_^2^) + (0.0577*P*)^2^] where *P* = (*F*_o_^2^ + 2*F*_c_^2^)/3
4421 reflections	(Δ/σ)_max_ < 0.001
200 parameters	Δρ_max_ = 1.08 e Å^−^^3^
0 restraints	Δρ_min_ = −0.54 e Å^−^^3^

### Special details

**Table:**

Experimental. Absorption correction: TWINABS2012/1 (Bruker, 2012) was used for absorption correction. For component 1: wR2(int) was 0.0813 before and 0.0454 after correction. The Ratio of minimum to maximum transmission is 0.77. Final HKLF 4 output contains 11962 reflections, Rint = 0.0892 (2973 with I > 3sig(I), Rint = 0.0335)
Geometry. All esds (except the esd in the dihedral angle between two l.s. planes) are estimated using the full covariance matrix. The cell esds are taken into account individually in the estimation of esds in distances, angles and torsion angles; correlations between esds in cell parameters are only used when they are defined by crystal symmetry. An approximate (isotropic) treatment of cell esds is used for estimating esds involving l.s. planes.
Refinement. Refined as a 2-component twin.

### Fractional atomic coordinates and isotropic or equivalent isotropic displacement parameters (Å^2^)

**Table:**

	*x*	*y*	*z*	*U*_iso_*/*U*_eq_	
Co1	0.61239 (8)	0.48208 (7)	0.64530 (5)	0.0137 (2)	
Si1	0.40656 (17)	0.25144 (14)	0.70655 (12)	0.0174 (3)	
Si2	0.71315 (17)	0.21357 (14)	0.66099 (12)	0.0167 (3)	
Si3	0.94365 (16)	0.68550 (14)	0.74896 (11)	0.0165 (3)	
Si4	0.68061 (16)	0.70994 (14)	0.84538 (11)	0.0165 (3)	
Na1	0.4185 (2)	0.27679 (19)	0.44187 (16)	0.0222 (5)	
O1	0.5000	0.5000	0.5000	0.0187 (11)	
N1	0.5621 (5)	0.2967 (4)	0.6570 (3)	0.0165 (9)	
N2	0.7424 (5)	0.6528 (4)	0.7365 (3)	0.0162 (9)	
C1	0.3045 (6)	0.3891 (5)	0.7230 (4)	0.0248 (13)	
H1A	0.2812	0.4278	0.6546	0.037*	
H1B	0.3762	0.4550	0.7884	0.037*	
H1C	0.2026	0.3571	0.7348	0.037*	
C2	0.4707 (7)	0.1928 (6)	0.8440 (5)	0.0312 (14)	
H2A	0.5074	0.1113	0.8336	0.047*	
H2B	0.3784	0.1780	0.8705	0.047*	
H2C	0.5600	0.2583	0.9001	0.047*	
C3	0.2418 (6)	0.1205 (5)	0.6018 (5)	0.0300 (14)	
H3A	0.1989	0.1514	0.5304	0.045*	
H3B	0.1540	0.0979	0.6315	0.045*	
H3C	0.2861	0.0435	0.5886	0.045*	
C4	0.6502 (7)	0.0351 (5)	0.6561 (5)	0.0255 (13)	
H4A	0.6269	0.0212	0.7247	0.038*	
H4B	0.7383	−0.0080	0.6517	0.038*	
H4C	0.5523	−0.0014	0.5895	0.038*	
C5	0.8974 (6)	0.2721 (6)	0.7914 (4)	0.0272 (14)	
H5A	0.9424	0.3638	0.7950	0.041*	
H5B	0.9791	0.2206	0.7893	0.041*	
H5C	0.8674	0.2625	0.8582	0.041*	
C6	0.7720 (6)	0.2294 (5)	0.5334 (4)	0.0233 (13)	
H6A	0.6872	0.1745	0.4663	0.035*	
H6B	0.8754	0.2018	0.5441	0.035*	
H6C	0.7843	0.3203	0.5232	0.035*	
C7	0.9702 (6)	0.5853 (5)	0.6277 (4)	0.0239 (13)	
H7A	0.9660	0.4953	0.6399	0.036*	
H7B	1.0758	0.6205	0.6222	0.036*	
H7C	0.8830	0.5874	0.5574	0.036*	
C8	1.0834 (6)	0.6525 (5)	0.8841 (4)	0.0249 (13)	
H8A	1.1074	0.7271	0.9448	0.037*	
H8B	1.1849	0.6385	0.8744	0.037*	
H8C	1.0310	0.5747	0.9038	0.037*	
C9	1.0216 (6)	0.8588 (5)	0.7425 (5)	0.0248 (13)	
H9A	0.9589	0.8788	0.6697	0.037*	
H9B	1.1367	0.8712	0.7503	0.037*	
H9C	1.0100	0.9168	0.8041	0.037*	
C10	0.6827 (6)	0.5923 (5)	0.9438 (4)	0.0242 (13)	
H10A	0.6380	0.5039	0.9000	0.036*	
H10B	0.6165	0.6131	0.9893	0.036*	
H10C	0.7949	0.5982	0.9939	0.036*	
C11	0.4724 (6)	0.7420 (5)	0.7848 (4)	0.0237 (13)	
H11A	0.4780	0.8203	0.7508	0.036*	
H11B	0.4269	0.7550	0.8450	0.036*	
H11C	0.4027	0.6677	0.7267	0.036*	
C12	0.8079 (6)	0.8685 (5)	0.9344 (4)	0.0232 (13)	
H12A	0.9202	0.8588	0.9700	0.035*	
H12B	0.7642	0.8948	0.9933	0.035*	
H12C	0.8060	0.9350	0.8868	0.035*	

### Atomic displacement parameters (Å^2^)

**Table:**

	*U*^11^	*U*^22^	*U*^33^	*U*^12^	*U*^13^	*U*^23^
Co1	0.0137 (4)	0.0132 (4)	0.0111 (4)	0.0022 (3)	0.0014 (3)	0.0003 (3)
Si1	0.0189 (8)	0.0167 (8)	0.0177 (8)	0.0036 (6)	0.0077 (6)	0.0030 (6)
Si2	0.0149 (7)	0.0165 (8)	0.0177 (8)	0.0037 (6)	0.0041 (6)	0.0032 (6)
Si3	0.0142 (7)	0.0180 (8)	0.0137 (7)	0.0021 (6)	0.0015 (6)	0.0007 (6)
Si4	0.0153 (7)	0.0170 (8)	0.0140 (7)	0.0007 (6)	0.0034 (6)	−0.0014 (6)
Na1	0.0268 (12)	0.0176 (12)	0.0171 (11)	0.0030 (9)	0.0018 (9)	0.0027 (9)
O1	0.022 (3)	0.017 (3)	0.014 (3)	0.002 (2)	0.003 (2)	0.001 (2)
N1	0.015 (2)	0.017 (2)	0.015 (2)	0.0014 (18)	0.0044 (18)	0.0006 (18)
N2	0.015 (2)	0.020 (3)	0.012 (2)	0.0036 (19)	0.0025 (17)	0.0016 (18)
C1	0.024 (3)	0.020 (3)	0.028 (3)	0.002 (2)	0.012 (2)	−0.004 (2)
C2	0.044 (4)	0.028 (4)	0.030 (3)	0.011 (3)	0.020 (3)	0.010 (3)
C3	0.028 (3)	0.024 (3)	0.038 (4)	0.002 (3)	0.017 (3)	−0.007 (3)
C4	0.028 (3)	0.023 (3)	0.030 (3)	0.011 (3)	0.013 (3)	0.008 (2)
C5	0.023 (3)	0.032 (4)	0.022 (3)	0.009 (3)	0.002 (2)	0.001 (2)
C6	0.025 (3)	0.018 (3)	0.021 (3)	0.006 (2)	0.002 (2)	−0.002 (2)
C7	0.021 (3)	0.022 (3)	0.027 (3)	0.003 (2)	0.009 (2)	0.000 (2)
C8	0.021 (3)	0.031 (4)	0.019 (3)	0.006 (3)	0.003 (2)	0.002 (2)
C9	0.018 (3)	0.025 (3)	0.027 (3)	−0.003 (2)	0.008 (2)	−0.004 (2)
C10	0.024 (3)	0.022 (3)	0.023 (3)	0.002 (2)	0.006 (2)	0.000 (2)
C11	0.027 (3)	0.018 (3)	0.027 (3)	0.005 (2)	0.009 (2)	0.005 (2)
C12	0.024 (3)	0.022 (3)	0.023 (3)	0.002 (2)	0.012 (2)	−0.003 (2)

### Geometric parameters (Å, º)

**Table:**

Co1—Na1^i^	2.918 (2)	C2—H2A	0.9800
Co1—O1	1.8398 (9)	C2—H2B	0.9800
Co1—N1	1.977 (4)	C2—H2C	0.9800
Co1—N2	1.980 (4)	C3—H3A	0.9800
Si1—N1	1.721 (4)	C3—H3B	0.9800
Si1—C1	1.861 (5)	C3—H3C	0.9800
Si1—C2	1.865 (6)	C4—H4A	0.9800
Si1—C3	1.869 (5)	C4—H4B	0.9800
Si2—N1	1.709 (4)	C4—H4C	0.9800
Si2—C4	1.872 (6)	C5—H5A	0.9800
Si2—C5	1.866 (5)	C5—H5B	0.9800
Si2—C6	1.874 (5)	C5—H5C	0.9800
Si3—Na1^i^	3.458 (3)	C6—H6A	0.9800
Si3—N2	1.717 (4)	C6—H6B	0.9800
Si3—C7	1.867 (5)	C6—H6C	0.9800
Si3—C8	1.872 (5)	C7—H7A	0.9800
Si3—C9	1.877 (6)	C7—H7B	0.9800
Si4—Na1^i^	3.490 (3)	C7—H7C	0.9800
Si4—N2	1.727 (4)	C8—H8A	0.9800
Si4—C10	1.863 (6)	C8—H8B	0.9800
Si4—C11	1.862 (5)	C8—H8C	0.9800
Si4—C12	1.870 (5)	C9—H9A	0.9800
Na1—Co1^i^	2.918 (2)	C9—H9B	0.9800
Na1—Si3^i^	3.458 (3)	C9—H9C	0.9800
Na1—Si4^i^	3.490 (3)	C10—H10A	0.9800
Na1—O1	2.314 (2)	C10—H10B	0.9800
Na1—N1	2.579 (4)	C10—H10C	0.9800
Na1—N2^i^	2.523 (4)	C11—H11A	0.9800
O1—Co1^i^	1.8399 (9)	C11—H11B	0.9800
O1—Na1^i^	2.314 (2)	C11—H11C	0.9800
N2—Na1^i^	2.523 (4)	C12—H12A	0.9800
C1—H1A	0.9800	C12—H12B	0.9800
C1—H1B	0.9800	C12—H12C	0.9800
C1—H1C	0.9800		
			
O1—Co1—Na1^i^	52.46 (5)	Si1—C1—H1B	109.5
O1—Co1—N1	108.39 (12)	Si1—C1—H1C	109.5
O1—Co1—N2	110.26 (13)	H1A—C1—H1B	109.5
N1—Co1—Na1^i^	159.62 (12)	H1A—C1—H1C	109.5
N1—Co1—N2	141.35 (17)	H1B—C1—H1C	109.5
N2—Co1—Na1^i^	58.31 (12)	Si1—C2—H2A	109.5
N1—Si1—C1	110.5 (2)	Si1—C2—H2B	109.5
N1—Si1—C2	114.4 (2)	Si1—C2—H2C	109.5
N1—Si1—C3	111.2 (2)	H2A—C2—H2B	109.5
C1—Si1—C2	108.3 (2)	H2A—C2—H2C	109.5
C1—Si1—C3	104.4 (2)	H2B—C2—H2C	109.5
C2—Si1—C3	107.5 (3)	Si1—C3—H3A	109.5
N1—Si2—C4	113.5 (2)	Si1—C3—H3B	109.5
N1—Si2—C5	113.1 (2)	Si1—C3—H3C	109.5
N1—Si2—C6	108.9 (2)	H3A—C3—H3B	109.5
C4—Si2—C6	106.2 (2)	H3A—C3—H3C	109.5
C5—Si2—C4	105.5 (3)	H3B—C3—H3C	109.5
C5—Si2—C6	109.2 (3)	Si2—C4—H4A	109.5
N2—Si3—Na1^i^	43.99 (14)	Si2—C4—H4B	109.5
N2—Si3—C7	109.4 (2)	Si2—C4—H4C	109.5
N2—Si3—C8	113.4 (2)	H4A—C4—H4B	109.5
N2—Si3—C9	113.5 (2)	H4A—C4—H4C	109.5
C7—Si3—Na1^i^	86.99 (17)	H4B—C4—H4C	109.5
C7—Si3—C8	108.6 (2)	Si2—C5—H5A	109.5
C7—Si3—C9	105.5 (3)	Si2—C5—H5B	109.5
C8—Si3—Na1^i^	157.06 (18)	Si2—C5—H5C	109.5
C8—Si3—C9	106.0 (3)	H5A—C5—H5B	109.5
C9—Si3—Na1^i^	84.84 (18)	H5A—C5—H5C	109.5
N2—Si4—Na1^i^	43.12 (14)	H5B—C5—H5C	109.5
N2—Si4—C10	111.6 (2)	Si2—C6—H6A	109.5
N2—Si4—C11	109.2 (2)	Si2—C6—H6B	109.5
N2—Si4—C12	114.5 (2)	Si2—C6—H6C	109.5
C10—Si4—Na1^i^	141.68 (18)	H6A—C6—H6B	109.5
C10—Si4—C12	106.4 (2)	H6A—C6—H6C	109.5
C11—Si4—Na1^i^	69.07 (18)	H6B—C6—H6C	109.5
C11—Si4—C10	110.1 (2)	Si3—C7—H7A	109.5
C11—Si4—C12	104.8 (2)	Si3—C7—H7B	109.5
C12—Si4—Na1^i^	110.76 (19)	Si3—C7—H7C	109.5
Co1^i^—Na1—Si3^i^	58.20 (6)	H7A—C7—H7B	109.5
Co1^i^—Na1—Si4^i^	57.47 (6)	H7A—C7—H7C	109.5
Si3^i^—Na1—Si4^i^	51.16 (5)	H7B—C7—H7C	109.5
O1—Na1—Co1^i^	39.08 (4)	Si3—C8—H8A	109.5
O1—Na1—Si3^i^	90.23 (8)	Si3—C8—H8B	109.5
O1—Na1—Si4^i^	94.36 (8)	Si3—C8—H8C	109.5
O1—Na1—N1	78.30 (12)	H8A—C8—H8B	109.5
O1—Na1—N2^i^	80.67 (12)	H8A—C8—H8C	109.5
N1—Na1—Co1^i^	117.16 (12)	H8B—C8—H8C	109.5
N1—Na1—Si3^i^	139.95 (12)	Si3—C9—H9A	109.5
N1—Na1—Si4^i^	165.71 (12)	Si3—C9—H9B	109.5
N2^i^—Na1—Co1^i^	41.89 (10)	Si3—C9—H9C	109.5
N2^i^—Na1—Si3^i^	28.21 (10)	H9A—C9—H9B	109.5
N2^i^—Na1—Si4^i^	27.90 (9)	H9A—C9—H9C	109.5
N2^i^—Na1—N1	155.82 (15)	H9B—C9—H9C	109.5
Co1—O1—Co1^i^	180.0	Si4—C10—H10A	109.5
Co1—O1—Na1^i^	88.46 (7)	Si4—C10—H10B	109.5
Co1—O1—Na1	91.54 (7)	Si4—C10—H10C	109.5
Co1^i^—O1—Na1^i^	91.54 (7)	H10A—C10—H10B	109.5
Co1^i^—O1—Na1	88.46 (7)	H10A—C10—H10C	109.5
Na1—O1—Na1^i^	180.00 (3)	H10B—C10—H10C	109.5
Co1—N1—Na1	81.02 (15)	Si4—C11—H11A	109.5
Si1—N1—Co1	116.1 (2)	Si4—C11—H11B	109.5
Si1—N1—Na1	104.36 (18)	Si4—C11—H11C	109.5
Si2—N1—Co1	115.7 (2)	H11A—C11—H11B	109.5
Si2—N1—Si1	124.7 (3)	H11A—C11—H11C	109.5
Si2—N1—Na1	101.38 (18)	H11B—C11—H11C	109.5
Co1—N2—Na1^i^	79.79 (14)	Si4—C12—H12A	109.5
Si3—N2—Co1	115.9 (2)	Si4—C12—H12B	109.5
Si3—N2—Si4	121.2 (2)	Si4—C12—H12C	109.5
Si3—N2—Na1^i^	107.8 (2)	H12A—C12—H12B	109.5
Si4—N2—Co1	114.6 (2)	H12A—C12—H12C	109.5
Si4—N2—Na1^i^	108.98 (19)	H12B—C12—H12C	109.5
Si1—C1—H1A	109.5		
			
Na1^i^—Co1—O1—Na1	179.999 (1)	C5—Si2—N1—Na1	151.5 (2)
Na1^i^—Si3—N2—Co1	87.1 (2)	C6—Si2—N1—Co1	−55.6 (3)
Na1^i^—Si3—N2—Si4	−126.4 (4)	C6—Si2—N1—Si1	146.4 (3)
Na1^i^—Si4—N2—Co1	−87.2 (2)	C6—Si2—N1—Na1	29.8 (2)
Na1^i^—Si4—N2—Si3	125.9 (4)	C7—Si3—N2—Co1	23.4 (3)
N1—Co1—O1—Na1^i^	172.03 (13)	C7—Si3—N2—Si4	170.0 (3)
N1—Co1—O1—Na1	−7.97 (13)	C7—Si3—N2—Na1^i^	−63.6 (3)
N2—Co1—O1—Na1	171.82 (13)	C8—Si3—N2—Co1	−98.0 (3)
N2—Co1—O1—Na1^i^	−8.18 (13)	C8—Si3—N2—Si4	48.5 (4)
C1—Si1—N1—Co1	6.4 (3)	C8—Si3—N2—Na1^i^	174.9 (2)
C1—Si1—N1—Si2	164.3 (3)	C9—Si3—N2—Co1	140.9 (2)
C1—Si1—N1—Na1	−80.6 (2)	C9—Si3—N2—Si4	−72.6 (3)
C2—Si1—N1—Co1	−116.1 (3)	C9—Si3—N2—Na1^i^	53.8 (3)
C2—Si1—N1—Si2	41.8 (4)	C10—Si4—N2—Co1	57.1 (3)
C2—Si1—N1—Na1	157.0 (2)	C10—Si4—N2—Si3	−89.8 (3)
C3—Si1—N1—Co1	121.8 (3)	C10—Si4—N2—Na1^i^	144.3 (2)
C3—Si1—N1—Si2	−80.3 (3)	C11—Si4—N2—Co1	−64.8 (3)
C3—Si1—N1—Na1	34.9 (3)	C11—Si4—N2—Si3	148.2 (3)
C4—Si2—N1—Co1	−173.7 (2)	C11—Si4—N2—Na1^i^	22.4 (3)
C4—Si2—N1—Si1	28.3 (4)	C12—Si4—N2—Co1	178.0 (2)
C4—Si2—N1—Na1	−88.3 (2)	C12—Si4—N2—Si3	31.1 (4)
C5—Si2—N1—Co1	66.1 (3)	C12—Si4—N2—Na1^i^	−94.8 (3)
C5—Si2—N1—Si1	−92.0 (3)		

Symmetry code: (i) −*x*+1, −*y*+1, −*z*+1.

## References

[bb1] Blakemore, J. D., Crabtree, R. H. & Brudvig, G. W. (2015). *Chem. Rev.* **115**, 12974–13005. 10.1021/acs.chemrev.5b00122 26151088

[bb2] Bruker (2012). *TWINABS*. Bruker AXS Inc., Madison, Wisconsin, USA.

[bb3] Bruker (2013). *SAINT*. Bruker AXS Inc., Madison, Wisconsin, USA.

[bb4] Bruker (2014). *APEX2*. Bruker AXS Inc., Madison, Wisconsin, USA.

[bb5] Bruker (2015). *APEX3*. Bruker AXS Inc., Madison, Wisconsin, USA.

[bb6] Clark, N. M., García-Álvarez, P., Kennedy, A. R., O’Hara, C. T. & Robertson, G. M. (2009). *Chem. Commun.* pp. 5835–5837. 10.1039/b908722b 19787113

[bb7] Dolomanov, O. V., Bourhis, L. J., Gildea, R. J., Howard, J. A. K. & Puschmann, H. (2009). *J. Appl. Cryst.* **42**, 339–341.

[bb8] Driess, M., Pritzkow, H., Skipinski, M. & Winkler, U. (1997). *Organometallics*, **16**, 5108–5112.

[bb9] Forbes, G. C., Kennedy, A. R., Mulvey, R. E., Rowlings, R. B., Clegg, W., Liddle, S. T. & Wilson, C. C. (2000). *Chem. Commun.* pp. 1759–1760.

[bb10] Groom, C. R., Bruno, I. J., Lightfood, M. P. & Ward, S. C. (2016). *Acta Cryst.* B**72**, 171–179. 10.1107/S2052520616003954 PMC4822653 27048719

[bb11] Hansen, C. B., Jordan, R. F. & Hillhouse, G. H. (2015). *Inorg. Chem.* **54**, 4603–4610. 10.1021/ic502670x 25938547

[bb12] Heering, C., Boldog, I., Vasylyeva, V., Sanchiz, J. & Janiak, C. (2013). *CrystEngComm*, **15**, 9757–9768.

[bb13] Kärkäs, M. D., Verho, O., Johnston, E. V. & Åkermark, B. (2014). *Chem. Rev.* **114**, 11863–12001. 10.1021/cr400572f 25354019

[bb14] Kennedy, A. R., Klett, J., Mulvey, R. E., Newton, S. & Wright, D. S. (2008). *Chem. Commun.* pp. 308–310. 10.1039/b714880a 18399190

[bb15] Kennedy, A. R., MacLellan, J. G. & Mulvey, R. E. (2003). *Acta Cryst.* C**59**, m302–m303. 10.1107/s0108270103011727 12909749

[bb16] Kennedy, A. R., Mulvey, R. E., Roberts, B. A., Rowlings, R. B. & Raston, C. L. (1999). *Chem. Commun.* pp. 353–354.

[bb17] Kennedy, A. R., Mulvey, R. E. & Rowlings, R. B. (1998). *Angew. Chem. Int. Ed.* **37**, 3180–3183. 10.1002/(SICI)1521-3773(19981204)37:22<3180::AID-ANIE3180>3.0.CO;2-D 29711337

[bb18] Li, Z.-Y., Dai, Y., Zhang, H., Zhu, J., Zhang, J.-J., Liu, S.-Q. & Duan, C.-Y. (2014). *Eur. J. Inorg. Chem.* pp. 384–391.

[bb19] Lu, X.-H., Ma, M.-T., Yao, Y.-M., Zhang, Y. & Shen, Q. (2010). *Inorg. Chem. Commun.* **13**, 1566–1568.

[bb20] Macrae, C. F., Edgington, P. R., McCabe, P., Pidcock, E., Shields, G. P., Taylor, R., Towler, M. & van de Streek, J. (2006). *J. Appl. Cryst.* **39**, 453–457.

[bb21] Matsubara, K., Sueyasu, T., Esaki, M., Kumamoto, A., Nagao, S., Yamamoto, H., Koga, Y., Kawata, S. & Matsumoto, T. (2012). *Eur. J. Inorg. Chem.* pp. 3079–3086.

[bb22] Mulvey, R. (2006). *Organometallics*, **25**, 1060–1075.

[bb23] Mulvey, R. E., Blair, V. L., Clegg, W., Kennedy, A. R., Klett, J. & Russo, L. (2010). *Nat. Chem.* **2**, 588–591. 10.1038/nchem.667 20571579

[bb24] Przyojski, J. A., Arman, H. D. & Tonzetich, Z. J. (2013). *Organometallics*, **32**, 723–732. 10.1021/om400630q PMC3931010 24567662

[bb25] Reger, D. L., Pascui, A. E., Foley, E. A., Smith, M. D., Jezierska, J. & Ozarowski, A. (2014). *Inorg. Chem.* **53**, 1975–1988. 10.1021/ic4017905 24479509

[bb26] Sarazin, Y., Coles, S. J., Hughes, D. L., Hursthouse, M. B. & Bochmann, M. (2006). *Eur. J. Inorg. Chem.* pp. 3211–3220.

[bb27] Sheldrick, G. M. (2008). *CELL_NOW*. University of Göttingen, Germany.

[bb28] Sheldrick, G. M. (2015*a*). *Acta Cryst.* A**71**, 3–8.

[bb29] Sheldrick, G. M. (2015*b*). *Acta Cryst.* C**71**, 3–8.

[bb30] Sumner, C. E. & Steinmetz, G. R. (1985). *J. Am. Chem. Soc.* **107**, 6124–6126.

[bb31] Wendelstorf, C. & Krämer, R. (1997). *Angew. Chem. Int. Ed. Engl.* **36**, 2791–2793.

[bb32] Wu, J., Pan, X., Tang, N. & Lin, C.-C. (2010). *Inorg. Chem.* **49**, 5362–5364. 10.1021/ic1006555 20491488

